# Does Body Mass Index Impact Outcomes in Patients Undergoing Minimally Invasive Mitral Valve Surgery?

**DOI:** 10.3390/medicina61050903

**Published:** 2025-05-16

**Authors:** Mariafrancesca Fiorentino, Elisa Mikus, Diego Sangiorgi, Alberto Tripodi, Simone Calvi, Elena Tenti, Antonino Costantino, Carlo Savini

**Affiliations:** 1 Division of Cardiac Surgery, Cardio-Vascular Surgery Department, Maria Cecilia Hospital, GVM Care & Research, 48033 Cotignola, Italy; elisamikus@yahoo.it (E.M.); dsangiorgi@gvmnet.it (D.S.); atripodi@gvmnet.it (A.T.); scalvi@gvmnet.it (S.C.); etenti@gvmnet.it (E.T.); antonino@costantinorc.com (A.C.); carlo.savini@unibo.it (C.S.); 2 Department of Experimental, Diagnostic and Specialty Medicine (DIMES), University of Bologna, 40126 Bologna, Italy

**Keywords:** body mass index, mitral valve surgery, minimally invasive cardiac surgery

## Abstract

*Background and Objectives:* This study examines the impact of Body Mass Index (BMI) on outcomes after mitral valve surgery via right minithoracotomy, an approach that may reduce wound complications in obese patients. *Materials and Methods:* Between January 2010 and December 2024, 1773 adult patients underwent minimally invasive mitral valve surgery at our institution. They were categorized into three groups: normal weight (BMI < 25, *n* = 942), overweight (BMI 25–30, *n* = 661), and obese (BMI > 30, *n* = 170). *Results:* The three groups exhibited significant differences, with a higher prevalence of hypertension, dyslipidemia, and diabetes (*p* < 0.001) in overweight and obese patients. Further-more, they had a greater incidence of preoperative atrial fibrillation (*p* < 0.001), prior stroke (*p* = 0.023), chronic obstructive pulmonary disease (*p* = 0.002), and elevated preoperative creatinine levels (*p* < 0.001). and their euroscore II was significantly higher (*p* = 0.040). In-hospital mortality and major complications were similar across groups, except for drainage output in the first 24 h (*p* = 0.002) and ICU stay (*p* = 0.022), both resulting higher in the overweight and obese patients. We employed inverse probability of treatment weighting (IPTW) to create three well-matched groups. Following IPTW, postoperative outcomes remained comparable across groups. However, obese patients exhibited a higher incidence of postoperative atrial fibrillation (*p* = 0.037) and required pacemaker implantation more frequently (*p* < 0.001). *Conclusions:* Our findings suggest that obesity does not increase the risk of mortality or major adverse events after minimally in-vasive mitral valve surgery. This approach may offer a less invasive alternative for obese patients, potentially reducing the risk of wound complications associated with conventional surgery.

## 1. Introduction

Obesity is widely recognized as a chronic disease that significantly contributes to global morbidity and mortality rates. In fact, it has been shown that in white adults, overweight and obesity (and possibly underweight) are associated with increased all-cause mortality [1]. More recently, the associations of both overweight and obesity with higher all-cause mortality have been found to be broadly consistent in four continents, with a log-linear increase in all-cause mortality with body mass index (BMI) values > 25 kg/m^2^ [2]. Notably, 67.5% of deaths associated with a high BMI are attributed to cardiovascular disease (CVD) [3]. Beyond its role in promoting well-established cardiovascular (CV) risk factors—such as type 2 diabetes mellitus (T2DM), dyslipidemia, elevated blood pressure, and arterial hypertension—obesity also exerts direct detrimental effects on cardiac structure and function. These effects contribute to the onset of both atherosclerotic and non-atherosclerotic forms of CVD, independent of other CV risk factors [4]. In cardiovascular surgery, the impact of obesity in increasing the risk of mortality and postoperative complications remains debated. In fact, there are studies [5] where obesity is associated with a lower risk of postoperative mortality, thus illustrating what is termed “the obesity paradox” [6]. However, other authors report that a BMI > 30 represents an independent risk factor for 30-day mortality, likely mediated by a longer duration of mechanical ventilation [7]. What is certain is that obesity, together with insulin-dependent diabetes and internal mammary artery grafting (especially bilaterally), increases the risk of sternal infection [8], which leads to prolonged hospital stays and higher mortality in these patients [9]. On the other hand, cardiovascular surgery is evolving toward the increasing use of minimally invasive techniques that spare the sternum, reduce the incidence of postoperative complications, including wound infections, and are becoming the standard of care for the treatment of certain conditions such as mitral valve disease [10]. The possibility of reducing the incidence of wound complications makes the use of these techniques particularly interesting in the obese patient population. This study aims to address a notable gap in the current literature by investigating the impact of BMI on outcomes after mitral valve surgery via right minithoracotomy. While existing research has explored aspects of minimally invasive mitral valve surgery and the influence of obesity on cardiac surgery outcomes, a specific analysis focusing on the interplay between BMI and outcomes following this particular surgical approach remains limited. Therefore, this research seeks to provide novel insights into this under-examined area, potentially informing clinical practice and improving patient management in obese individuals undergoing this procedure

## 2. Materials and Methods

### 2.1. Study Population

All adult patients (age > 18 years old) who underwent isolated or combined mitral valve surgery through a right-sided mini-thoracotomy were included in the present study. No sample size calculation was performed, and all available patients were included in this study. The decision to use a minimally invasive approach was left to the surgeon’s preference. From January 2010 to October 2024, a total of 1773 adult patients underwent minimally invasive mitral valve surgery at our institution and represent our study population. They were categorized into three groups, according to the definition given by the World Health Organization: normal weight (BMI < 25, n = 942), overweight (BMI 25–30, n = 661), and obese (BMI > 30, n = 170); since there were only 42 underweight patients (BMI < 18.5), they were combined with the normal weight group; obese patients in obesity class II (35–39.9) were 23, while 9 where in obesity class III (>40). This study was conducted in accordance with the Declaration of Helsinki, and the protocol was approved by the Romagna Ethics Committee on 20 November 2019 (Prot.9689/2019 I.5/186). Individual informed consent was waived due to the retrospective nature of the data collected. We retrospectively analyzed preoperative, intra-operative, and postoperative clinical data of all patients using medical records, and every possible effort was made to reduce missing information. Only complete cases were analyzed. The primary endpoints were in-hospital mortality, defined as any death occurring before discharge from the index hospitalization, the occurrence of any postoperative adverse event, and conversion to full sternotomy. Additionally, patient follow-up was performed using phone calls and physician visits to assess medium and long-term survival and freedom from reoperations.

### 2.2. Operative Strategy

Surgical indications were determined based on the guidelines from the American College of Cardiology/American Heart Association and the European Society of Cardiology/European Association of Cardiothoracic Surgery relevant to each period [11,12,13,14,15,16]. All patients received total intravenous anesthesia and selective intubation with a double-lumen endotracheal tube. Transesophageal echocardiography was employed in all heart valve surgeries at our institution to monitor heart and valve function both before and after the procedure. The femoral vessels were exposed through a 3-cm incision, and the femoral artery and vein were cannulated using the Seldinger technique under transesophageal echocardiographic guidance. In female patients, a 4- to 6-cm skin incision was made at the inframammary fold, while in male patients, it was made at the level of the 4th right intercostal space. Thoracotomy was performed at the level of the fourth intercostal space. Two additional ports were placed in the fourth and sixth intercostal spaces for the intracardiac suction line, carbon dioxide delivery, and thoracoscope insertion. The pericardium was incised 2 to 3 cm above the phrenic nerve, and retraction sutures were placed to pull it toward the right chest. Cardiopulmonary bypass (CPB) was initiated, and the patient was cooled to 30–32 °C. The aorta was cross-clamped using either a Chitwood clamp inserted through a separate 10-mm skin incision or a Cygnet clamp from within the thoracotomy. Hypothermic blood cardioplegia (St. Thomas with procaine) or Custodiol cardioplegia was delivered in an antegrade manner into the aortic root for cardiac protection. If tricuspid valve surgery was required, the caval veins were snared around the cannula before opening the right atrium, allowing adequate exposure of the tricuspid valve. Once cardioplegic arrest was achieved, the left atrium was opened, and the mitral valve was either repaired or replaced. Following this, the left atrium was closed, and the right atrium was opened for tricuspid valve repair if necessary. After right atrium closure, ventricular pacing wires were placed on the right ventricle, the aorta was declamped, and the patient was weaned from cardiopulmonary bypass in a standard manner. Postoperative management at our institution follows the same protocols as for patients undergoing surgery via full sternotomy. Specifically, upon arrival at the intensive care unit (ICU), patients are typically extubated on postoperative day (POD) 0 after evaluating thoracic drain output, level of consciousness, and gas exchange. They generally spend the first night in the ICU and the second night in a medium care unit (MCU) if clinical conditions permit. On POD 1, all patients are mobilized to an armchair. Thoracic drains are removed on POD 2, and patients are transferred to the ward, where they are fully mobilized. This approach typically allows for discharge home on POD 7.

### 2.3. Statistical Analysis

Continuous variables were assessed for normality using the Shapiro–Wilk test. As they did not follow a normal distribution, they were summarized as a median and interquartile range (Q1–Q3; 1st–3rd quartile) and compared using the Kruskal–Wallis test. Categorical variables were presented as absolute counts and percentages and analyzed with Fisher’s exact test. To adjust for BMI category imbalances, the inverse probability of treatment weighting (IPTW) method with covariate balancing propensity score (CBPS) was applied, using baseline characteristics to estimate weights. Balance across groups was evaluated using absolute standardized mean differences (ASMD), considering variables with ASMD < 0.2 as adequately balanced. After weighting, medians (Q1–Q3) and percentages were reported. Weighted univariate logistic regression models were used to analyze outcomes. All statistical analyses were conducted using R 4.4.0 (R Foundation for Statistical Computing, Vienna, Austria), with a significance threshold of *p* < 0.05.

## 3. Results

### 3.1. Patient Characteristics

Preoperative patient characteristics according to BMI are presented in Table 1. The median (Q1–Q3) age of our population was similar between the three groups, while both overweight and obese patients were more frequently male (*p* < 0.001), with a higher prevalence of the most common cardiovascular risk factors as hypertension, dyslipidemia, and diabetes (*p* < 0.001). In all the groups, the majority were in New York Heart Association (NYHA) class II or III at the time of surgery and the median (Q1–Q3) left ventricle ejection fraction was 60% (55–65). There were no significant differences in the percentage of patients suffering from active endocarditis (*p* = 0.088), pulmonary hypertension (*p* = 1.000), or having experienced previous cardiac surgery (*p* = 0.930). Conversely, overweight and obese patients showed a greater incidence of preoperative atrial fibrillation (*p* < 0.001), prior stroke (*p* = 0.023), chronic obstructive pulmonary disease (*p* = 0.002), and elevated preoperative creatinine levels (*p* < 0.001), along with a significantly higher EuroSCORE II (*p* = 0.040). As explained in the statistical analysis section, we applied the inverse probability of treatment weighting (IPTW) method with covariate balancing propensity score (CBPS) to adjust for BMI category imbalances, using baseline characteristics to estimate weights. After weighting, medians (Q1–Q3) and percentages are reported in Table 2.

### 3.2. In-Hospital Outcomes

**Figure 1:**
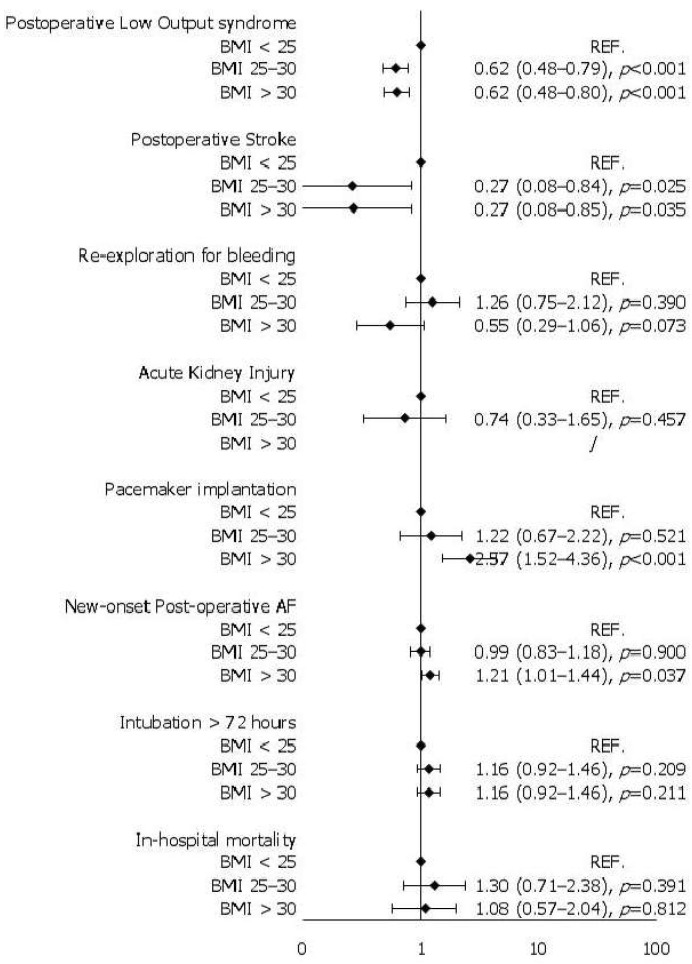
Univariable logistic regression models after IPTW.

Intraoperative and in-hospital outcomes before IPTW are summarized in Table 3. Before matching, the three groups were similar for cardiopulmonary bypass, cross-clamp time, and the need for associated procedures. However, overweight (BMI > 25) and obese patients (BMI > 30) more frequently underwent mitral valve replacement instead of repair (*p* = 0.006). As for in-hospital outcomes, we observed significant differences between the three groups only in drainage output in the first 24 h (*p* = 0.002) and ICU stay (*p* = 0.022), both of which were higher in overweight and obese patients. All the other postoperative outcomes analyzed were comparable in the three groups, including the incidence of cardiogenic shock requiring mechanical circulatory support. Weighted univariate logistic regression models demonstrated comparable in-hospital outcomes across the three groups (Figure 1), except for obese patients, who exhibited a higher incidence of postoperative atrial fibrillation (*p* = 0.037) and required pacemaker implantation more frequently (*p* < 0.001). On the other hand, both overweight and obese patients suffered less frequently from low output syndrome (*p* < 0.001) and postoperative stroke (*p* = 0.025) compared to the normal-weight population.

## 4. Discussion

In this paper, we present our fourteen-year experience with a minimally invasive approach to mitral valve surgery. Over the years, surgeons’ expertise has progressively increased, leading to the expanded adoption of this approach across all patient categories [10], including high-risk categories, such as elderly [17] and overweight and obese patients [18]. Indeed, the use of a minimally invasive approach may potentially improve postoperative outcomes in higher-risk patients by reducing surgical invasiveness and minimizing the overall physiological impact of the procedure. Specifically, regarding the overweight and obese population, these patients are at a higher risk of 30-day mortality, primarily due to the need for prolonged intubation [7], as well as an increased risk of wound dehiscence [8,9], leading to a longer hospital stay. These outcomes could potentially be influenced by the use of a minimally invasive approach. The potential impact of a minimally invasive approach on the outcomes of patients undergoing valve surgery has already been investigated in the literature. The existing evidence suggests that a minimally invasive approach to valve surgery holds promise for improved outcomes, particularly in obese patients who are at higher risk for complications with traditional sternotomy. Santana et al.’s 2011 study [18] demonstrated a significant reduction in postoperative complications, including renal failure, prolonged intubation, wound issues, and mortality, with minimally invasive surgery in obese patients compared to sternotomy. While Aljanadi et al.’s later research [19] found no significant difference in overall perioperative outcomes or long-term survival based on BMI in minimally invasive mitral valve surgery, and the Hannover group [20] observed higher rates of specific complications in obese patients undergoing MIMVS, both studies concluded that obesity should not be a barrier to offering this less invasive technique. These findings underscore the potential clinical benefit of adopting minimally invasive approaches in obese patients undergoing valve surgery. By potentially lowering morbidity and mortality, these techniques could lead to faster recovery times, reduced hospital stays, and improved quality of life in this challenging patient population. Further research specifically examining the impact of BMI on outcomes after mitral valve surgery via right minithoracotomy, as proposed in our study, will contribute crucial data to refine patient selection criteria and optimize the application of this promising surgical strategy in obese individuals.

Our results are partially consistent with those reported in the literature, with some key differences. First, we report a total of 1773 isolated or combined mitral valve surgeries over a 14-year period. Second, we categorized our population into three groups instead of two, including overweight patients (BMI between 25 and 30), whose characteristics and outcomes are typically considered similar to those of the normal-weight population. Upon categorizing our population into groups, we observed a significant imbalance, as overweight and obese patients had a higher prevalence of traditional cardiovascular risk factors (diabetes, hypertension, dyslipidemia) and a higher surgical risk profile. This was reflected in an increased incidence of COPD, previous atrial fibrillation, prior stroke, and higher creatinine levels, resulting in an elevated EuroSCORE II. However, despite the higher surgical risk profile, we did not identify any significant differences in terms of in-hospital mortality or major postoperative adverse events. Overweight and obese patients only exhibited greater chest drain output in the first 24 h and a longer intensive care unit stay compared to normal-weight patients, which could be attributed to the need for more frequent monitoring of respiratory exchanges, as it is more frequently compromised in obese patients both before and after surgery due to excess body weight. To balance our populations and adjust for preoperative differences, we applied the inverse probability of treatment weighting (IPTW) method with covariate balancing propensity score (CBPS). Following this adjustment, the three categories no longer differed based on preoperative variables, and postoperative outcomes were comparable across the three groups. This suggests that the differences observed in the unmatched population were not attributable to BMI variations but rather to the distinct preoperative risk profiles. However, in the weighted univariable analysis performed on the three populations post-IPTW, a BMI > 30 was identified as a risk factor for the development of postoperative atrial fibrillation (POAF) and the need for pacemaker implantation, an association not observed in the overweight population (BMI 25–30). The relationship between obesity and POAF has been previously investigated by Phan et al. [21], who demonstrated that obese patients had significantly higher odds of developing POAF compared to non-obese patients (*p* = 0.006). Furthermore, the occurrence of POAF was associated with an increased risk of stroke (*p* < 0.0001), 30-day mortality (*p* = 0.005), and respiratory complications (*p* < 0.00001). Proposed mechanisms underlying this increased susceptibility include left atrial stretch, diastolic dysfunction, and elevated plasma volume secondary to obesity, all of which may contribute to atrial vulnerability and the development of POAF [22]. Similarly, the increased incidence of pacemaker implantation in obese patients has also been reported in those undergoing transcatheter aortic valve implantation (TAVI) [23]. A potential explanation for this phenomenon is the presence of metaplastic and infiltrative changes affecting the sinus node, atrioventricular node, right bundle branch, and myocardium adjacent to the atrioventricular ring, which may contribute to cardiac conduction abnormalities [24]. However, whether these structural changes predispose patients undergoing TAVI or cardiac surgery to a high-degree atrioventricular block and subsequent pacemaker implantation remains uncertain [25]. Our findings indicate that in patients undergoing mitral valve surgery via a minimally invasive approach, obesity and overweight do not increase the risk of several major postoperative complications, including bleeding requiring re-exploration, acute kidney injury, prolonged intubation, in-hospital death, or wound breakdown. Intriguingly, our data even suggest a potential protective effect of overweight and moderate obesity against low cardiac output syndrome and stroke after this type of surgery—a phenomenon known as the ‘obesity paradox’ [5]. This implies that a higher BMI should not be considered a contraindication for minimally invasive mitral valve surgery. Clinically, this is significant because it suggests that even patients traditionally deemed high-risk due to their weight can safely benefit from this less invasive surgical option, with postoperative mortality and complication rates comparable to those of the general population and may even have a lower risk of postoperative stroke and low cardiac output syndrome. Importantly, they do not face a heightened risk of wound complications, a common concern in heavier patients undergoing traditional open-heart surgery. This advantage should encourage clinicians to consider and offer minimally invasive mitral valve surgery to a broader range of patients, including those with elevated BMI, potentially leading to comparable or even better outcomes without the increased wound risks associated with sternotomy.

### Limitations

The main limitation of this study is its retrospective nature. All data represent a single-center experience and the decision to use a minimally invasive approach was left to the surgeon’s discretion. Furthermore, the patients were not compared to those undergoing the same surgery through conventional sternotomy. Additionally, the surgical approach was chosen at the surgeon’s discretion without a randomization process, which introduces a potential selection bias. Finally, we do not have follow-up data, which would have further strengthened this study.

## 5. Conclusions

In our single-center experience, a minimally invasive approach through right-sided mini-thoracotomy has proven to be both a safe and feasible approach for mitral valve surgery, regardless of patient BMI. In our population, obesity was not associated with an increased risk of mortality or major adverse events following minimally invasive mitral valve surgery. Furthermore, the similar rates of wound dehiscence across all BMI groups suggest that this approach offers a safer alternative for obese patients by potentially reducing the wound complications typically seen with traditional sternotomy. These findings strongly support the safety and feasibility of minimally invasive mitral valve surgery in overweight and obese individuals, advocating for its increased consideration in this population to minimize morbidity associated with conventional open-heart surgery Therefore, minimally invasive mitral valve surgery represents a promising option for overweight and obese patients and should be further advocated.

## Figures and Tables

**Table 1:** Patient characteristics before IPTW.

	BMI < 25	BMI 25–30	BMI > 30	*p*	SMD
N	942	661	170		
Age, median (Q1–Q3)	64 (54, 74)	66 (57, 74)	67 (60, 73)	0.071	0.113
Male, n (%)	515 (54.7)	466 (70.5)	104 (61.2)	<0.001	0.220
Hypertension, n (%)	446 (47.3)	405 (61.3)	119 (70.0)	<0.001	0.313
Diabetes, n (%)	51 (5.4)	57 (8.6)	32 (18.8)	<0.001	0.282
Dyslipidemia, n (%)	321 (34.1)	271 (41.0)	81 (47.6)	<0.001	0.185
Smoke, n (%)	260 (27.6)	198 (30.0)	53 (31.2)	0.448	0.052
COPD, n (%)	33 (3.5)	40 (6.1)	16 (9.6)	0.002	0.167
Preoperative AF, n (%)	132 (14)	126 (19.1)	44 (25.9)	0.001	0.220
Preoperative pacemaker, n (%)	5 (0.5)	8 (1.2)	0 (0.0)	0.224	0.111
NYHA class, n (%)				0.017	0.216
1	242 (26.1)	146 (22.4)	23 (14.0)		
2	451 (48.7)	327 (50.2)	87 (53.0)		
3	210 (22.7)	166 (25.5)	50 (30.5)		
4	23 (2.5)	13 (2.0)	4 (2.4)		
LVEF, median (Q1–Q3)	60 (55, 65)	60 (55, 65)	60 (55, 65)	0.059	0.102
Active endocarditis, n (%)	30 (3.2)	10 (1.5)	5 (2.9)	0.088	0.074
Previous stroke, n (%)	8 (0.8)	14 (2.1)	5 (2.9)	0.023	0.104
Previous TIA, n (%)	3 (0.3)	10 (1.5)	3 (1.8)	0.011	0.096
Preoperative creatinine, median (Q1–Q3)	0.90 (0.78, 1.01)	0.97 (0.84, 1.10)	0.99 (0.87, 1.11)	<0.001	0.156
Pulmonary hypertension, n (%)	411 (43.7)	279 (42.2)	85 (50.0)	0.190	0.153
Previous cardiac surgery, n (%)	43 (4.6)	32 (4.9)	8 (4.8)	0.930	0.010
EuroSCORE Log, median (Q1–Q3)	2.88 (1.83, 6.00)	3.08 (1.72, 5.48)	3.73 (2.19, 5.96)	0.072	0.116
EuroSCORE II, median (Q1–Q3)	1.12 (0.75, 2.17)	1.10 (0.69, 1.86)	1.23 (0.79, 2.26)	0.040	0.081

COPD: chronic obstructive pulmonary disease; AF: atrial fibrillation; NYHA: New York Heart Association; LVEF: left ventricle ejection fraction; Q1–Q3: first-third quartile; TIA: transient ischemic attack.

**Table 2:** Patient characteristics after IPTW.

	BMI < 25	BMI 25–30	BMI > 30	SMD
N	1.789, 88	1.791, 46	1.781, 03	
Age, median (Q1–Q3)	65 (56, 74)	65 (56, 74)	66 (58, 73)	0.004
Male, n (%)	1094.0 (61.1)	1094.8 (61.1)	1088.4 (61.1)	<0.001
Hypertension, n (%)	1003.2 (56.0)	1004.2 (56.1)	994.9 (55.9)	0.003
Diabetes, n (%)	155.0 (8.7)	155.1 (8.7)	154.3 (8.7)	<0.001
Dyslipidemia, n (%)	687.0 (38.4)	687.6 (38.4)	681.4 (38.3)	0.002
Smoke, n (%)	503.0 (28.1)	503.7 (28.1)	498.1 (28.0)	0.002
COPD, n (%)	94.1 (5.3)	94.0 (5.3)	92.6 (5.3)	0.002
Preoperative AF, n (%)	309.3 (17.3)	303.3 (17.9)	333.8 (18.7)	0.106
Preoperative pacemaker, n (%)	8.9 (0.5)	10.4 (0.6)	0.0 (0.0)	0.073
NYHA class, n (%)				0.092
1	409.6 (23.3)	403.4 (22.9)	351.2 (20.1)	
2	862.3 (49.1)	865.4 (49.2)	954.8 (54.6)	
3	435.4 (24.8)	450.8 (25.6)	409.3 (23.4)	
4	49.6 (2.8)	38.9 (2.2)	32.8 (1.9)	
LVEF, median (Q1–Q3)	60 (55, 65)	60.00 (55, 65)	60 (55, 66)	0.003
Active endocarditis, n (%)	41.3 (2.3)	41.8 (2.3)	40.8 (2.3)	0.002
Previous stroke, n (%)	32.3 (1.8)	32.2 (1.8)	32.3 (1.8)	0.001
Previous TIA, n (%)	17.3 (1.0)	17.3 (1.0)	17.3 (1.0)	<0.001
Preoperative creatinine, median (Q1–Q3)	0.91 (0.80, 1.04)	0.93 (0.80, 1.08)	0.97 (0.87, 1.09)	0.001
Pulmonary hypertension, n (%)	804.8 (45.0)	770.6 (43.0)	913.9 (51.3)	0.151
Previous cardiac surgery, n (%)	79.4 (4.5)	79.3 (4.5)	77.0 (4.4)	0.004
EuroSCORE Log, median (Q1–Q3)	3.08 (1.83, 6.01)	3.13 (1.72, 5.82)	3.51 (2.08, 5.56)	0.006
EuroSCORE II, median (Q1–Q3)	1.16 (0.76, 2.33)	1.10 (0.69, 1.88)	1.07 (0.76, 1.97)	<0.001

COPD: chronic obstructive pulmonary disease; AF: atrial fibrillation; NYHA: New York Heart Association; LVEF: left ventricle ejection fraction; Q1–Q3: first-third quartile; TIA: transient ischemic attack.

**Table 3:** In-hospital outcomes before IPTW.

	BMI < 25	BMI 25–30	BMI > 30	*p*	SMD
N	942	661	170		
CPB time, median (Q1–Q3)	96 (66, 125)	100 (63, 131)	104 (76, 136)	0.067	0.110
Cross-clamp time median (Q1–Q3)	78 (52, 102)	82 (51, 107)	83 (60, 107)	0.130	0.098
Associated procedures, n (%)	248 (26.3)	189 (28.6)	46 (27.1)	0.605	0.034
Mitral valve replacement, n (%)	160 (17.0)	128 (19.4)	47 (27.6)	0.006	0.172
Postoperative low output syndrome, n (%)	75 (8.0)	40 (6.1)	11 (6.5)	0.341	0.050
Postoperative stroke, n (%)	7 (0.7)	1 (0.2)	1 (0.6)	0.183	0.060
Re-exploration for bleeding, n (%)	13 (1.4)	13 (2.0)	2 (1.2)	0.689	0.042
Acute kidney injury, n (%)	11 (1.2)	8 (1.2)	5 (2.9)	0.167	0.084
Postoperative RRT, n (%)	3 (0.3)	5 (0.8)	2 (1.2)	0.188	0.068
Pacemaker implantation, n (%)	10 (1.1)	8 (1.2)	5 (2.9)	0.128	0.090
New-onset post-operative AF, n (%)	146 (15.5)	102 (15.4)	31 (18.2)	0.634	0.050
Intubation > 72 h, n (%)	73 (7.7)	60 (9.1)	21 (12.4)	0.137	0.102
Wound complications, n (%)	33 (3.5)	18 (2.7)	3 (1.8)	0.625	0.096
Postoperative depsis, n (%)	4 (0.4)	3 (0.5)	3 (1.8)	0.125	0.086
Ventilation time, median (Q1–Q3)	6 (4, 10)	6 (4, 10)	6 (5, 11)	0.112	0.027
Bleeding in 24 h, median (Q1–Q3)	350 (250, 500)	400 (300, 550)	400 (250, 500)	0.002	0.131
In-hospital mortality, n (%)	10 (2.3)	10 (3.2)	1 (1.4)	0.703	0.082
ICU days, median (Q1–Q3)	2.0 (1.9, 2.0)	2.0 (1.9, 2.0)	2.0 (2.0, 3.0)	0.022	0.111
LOS days, median (Q1–Q3)	7.0 (7.0, 9.0)	7.0 (7.0, 8.0)	7.0 (7.0, 9.0)	0.058	0.050

IQR: interquartile range; RRT: renal replacement therapy; AF: atrial fibrillation; ICU: intensive care unit; LOS: length of stay.

## Data Availability

The data presented in this study are available on request from the corresponding author. The data are not publicly available due to Data Protection Directive 95/46/EC.
